# Prévalence de l’hépatite B chronique selon le statut sérologique VIH à Parakou au Bénin

**DOI:** 10.11604/pamj.2018.30.180.16117

**Published:** 2018-06-27

**Authors:** Salimanou Ariyoh Amidou, Comlan Albert Dovonou, Corine Houehanou, Arsène Amadohoué Kpangon, Rhonel Ahanhanzo-Glele, Julie Hounnouga Kpangon, Khadidjatou Saké Alassan, Attinsounon Cossi Angelo, Blaise Tchaou, Kabibou Salifou, Thierry Adoukonou, Djimon Marcel Zannou, Dismand Stephan Houinato

**Affiliations:** 1 Centre d’Information de Prospectives et de Conseils sur les IST/VIH/Sida, Parakou, Bénin; 2 Service de Médecine Interne, Centre Hospitalier Universitaire de Parakou, Bénin; 3 Laboratoire d’Epidémiologie des Maladies Chroniques et Neurologiques (LEMACEN), Faculté des Sciences de la Santé de Cotonou, Bénin; 4 Service des Urgences, Centre Hospitalier Universitaire de Parakou, Bénin; 5 Service de Gynécologie-Obstétrique, Centre Hospitalier Universitaire de Parakou, Bénin; 6 Clinique Universitaire de Médecine Interne, CNHU-HKM de Cotonou, Bénin

**Keywords:** VIH, Hépatite B, Prévalence, Parakou, Bénin, HIV, hepatitis B, prevalence, Parakou, Benin

## Abstract

**Introduction:**

La prévalence de l’hépatite B est très variable à travers les espaces géographiques et semble influencée par l’infection à VIH. La présente étude vise à comparer la prévalence de l’hépatite B en fonction du statut sérologique VIH en milieu hospitalier à Parakou au Bénin.

**Méthodes:**

Il s’agissait d’une étude transversale incluant les adultes de 18 ans et plus reçus au Centre Hospitalier Universitaire et Départemental de Parakou entre Mai 2011 et Juin 2012. Le diagnostic de l’hépatite B a été fait par la recherche de l’antigène HBs et celui du VIH par des tests rapides sur des prélèvements de sang veineux. Les données ont été analysées avec le logiciel EpiInfo. Un modèle de régression logistique multivariable a été développé pour explorer les facteurs associés à la présence de l’hépatite B.

**Résultats:**

Sur un total de 1516 sujets inclus, 744 étaient séropositifs au VIH. L’âge moyen était de 31,3 + 11,1 ans et 65,1% étaient des femmes. La prévalence de l’hépatite B dans l’ensemble de l’échantillon a été estimée à 13,9% [IC95: 12,2%-15,7%]. Cette prévalence était plus élevée chez les sujets séropositifs au VIH (16,9% vs 10,9%; p < 0,0006), mais la différence n’est cependant plus significative en analyse multivariée, en dehors du groupe des sujets originaires du Borgou/Alibori (p < 0,02). Une association constante a toutefois été observée entre la tranche d’âge de 24 à 44 ans (p < 0,03), le sexe masculin (p < 0,01), le niveau d’étude primaire (p < 0,02) et une prévalence élevée de l’hépatite B.

**Conclusion:**

La prévalence de l’hépatite B était plus élevée chez les sujets séropositifs au VIH. Elle est influencée par l’âge, le sexe, le niveau d’instruction et l’origine géographique.

## Abstract (en)

**Introduction:**

The prevalence of hepatitis B is very variable across geographic areas and seems to be influenced by HIV infection. This study aims to evaluate the impact of serologic HIV status on the overall prevalence of hepatitis B in a Hospital in Parakou, Benin.

**Methods:**

We conducted a cross-sectional study of adults aged 18 years old and over hospitalized in the Departmental University Hospital Center in Parakou between May 2011 and June 2012. The diagnosis of hepatitis B was made on the basis of rapid HBsAg tests while the diagnosis of HIV was made on the basis of rapid HIV tests using venous blood samples. Data were analyzed using EpiInfo software. Multivariate logistic regression model was implemented to investigate factors associated with hepatitis B.

**Results:**

Out of 1516 subjects included, 744 were HIV seropositive. The average age was 31.3 + 11.1 years and 65.1% were women. The prevalence rate of hepatitis B in the whole sample was 13.9% [CI95:12.2%-15.7%]. This prevalence was higher in HIV seropositive subjects (16.9% vs 10.9%; p < 0.0006), however there was not a more significant difference in multivariate analysis, except for the group of subjects from Borgou/Alibori (p < 0.02). A consistent association was observed between age group 24-44 years (p < 0.03), male sex (p < 0.01), primary school education (p < 0.02) and a high prevalence of hepatitis B.

**Conclusion:**

The prevalence of hepatitis B was higher in HIV seropositive subjects. This was influenced by age, sex, education level and geographical origin.

## Introduction

Dans le monde, il y avait 325 millions de personnes affectées par l’hépatite en 2015, dont 257 millions par l’hépatite B et 71 millions par l’hépatite C. Les virus B et C des hépatites sont responsables des mortalités les plus élevées parmi les cinq types d’hépatite [1]. La prevalence de l’hépatite B varie selon les Régions de l’OMS, la region africaine et le pacifique occidental ont le plus lourd fardeau avec les prévalences les plus élevée [2]. En Afrique, la prévalence du VHB est élevée avec des disparités selon les pays, les régions et en fonction des milieux de vie rural/urbain [3–5]. L’OMS en adoptant la stratégie mondiale de lutte contre l’hépatite B de 2016 à 2021 met un accent sur la recherche en son Orientation stratégique 1 qui visent à produire des données pour cibler les actions en mettant au point un système d’information stratégique solide afin de comprendre les épidémies d’hépatite virale et de cibler la riposte [6]. La connaissance de la situation particulière de chaque pays, de chaque région, de chaque groupe cible est donc primordiale pour adapter les stratégies de contrôle qui permettraient d’atteindre l’objectif d’élimination de l’hépatite B à travers l’accès aux soins et la vaccination. L’insuffisance de données épidémiologiques ralentit donc la riposte. Le présent travail visait à contribuer à combler le gap. Une précédente étude réalisée au Centre Hospitalier Universitaire et Départemental (CHUD) de Parakou avait estimé la prévalence brute de l’hépatite B au sein des personnes séropositives au VIH à 16,9% (IC95: 14,3%-19,9%) [7] plus élevée que celle décrite précédemment à Cotonou chez les mêmes cibles (11,2%) [8], mais sans différence significative. Il s’est cependant avéré nécessaire d’approfondir le sujet en comparant ces résultats à celle d’une population similaire de personnes séronégatives au VIH pour discriminer le rôle de l’espace géographique et ou de l’infection à VIH dans la distribution de la prévalence de l’infection à VIH. C’est dans ce cadre que se situait la présente étude qui visait à comparer la séroprévalence du VHB en fonction du statut VIH chez les patients vus en consultation externe au CHUD de Parakou et identifier les facteurs associés à cette co-infection.

## Méthodes

### Schéma et population d’étude

Il s’agissait d’une étude transversale comparative. Elle a inclus deux groupes. Le premier groupe était composé de l’ensemble des personnes séropositives au VIH suivies au CHUD de Parakou. Le second groupe était composé de l’ensemble des personnes âgées de 15 ans et plus, reçus en consultation externe dans les services des urgences, de médecine interne, de gynécologie-obstétrique, au centre de dépistage du VIH du CHUD de Parakou, et contrôlées séronégatives au VIH. Les personnes éligibles n’ayant pas donné leur consentement éclairé et écrit ont été exclus de l’étude.

### Echantillonnage et taille d’échantillon

Nous avons procédé à un échantillonnage aléatoire systématique. Ainsi, l’enquête a été proposée à tous les patients séropositifs au VIH reçus en consultation dans le service de Médecine durant la période de Mai 2011 à Avril 2012, et à toutes les personnes consultant dans les services de Médecine, Urgences et Gynécologie entre Février et Avril 2012. Ces périodes d’étude ont été déterminées en fonction de la taille de l’échantillon visée et du flux des patients dans les services concernés. La taille de l’échantillon a été calculée à l’aide du module STATCALC de EpiInfo sur la base des hypothèses suivantes: le type d’étude, un risque a de 5%, une puissance (1-ß) de 80% ; un ratio sujet séropositif au VIH/sujet séronégatifs au VIH = 1/1, une prévalence VHB attendue chez les sujets séronégatifs au VIH = 10%, un rapport de prévalence RP attendu de 1,5 (d’après une prévalence de co-infection VIH et VHB estimée par Barth [**3**]. La taille a été estimée à environ 1450 personnes soit 725 dans chaque groupe.

### Collecte des données

Elle s’est déroulée en 3 étapes pour tous les participants: l’entretien en face à face avec le patient pour collecter les données sociodémographiques et les antécédents; le dépouillement du dossier médical pour collecter les données cliniques et thérapeutiques; enfin l’analyse de sang veineux pour la recherche de l’Ag HBs et des anticorps anti VIH. Le diagnostic de l’infection à VIH a été fait selon les normes en vigueur au Bénin (Stratégie 2 de l’OMS). Elle a consisté en l’utilisation de deux tests: un test sensible (test rapide Determine^®^) et un test discriminant (Génie 2^®^, Bioline^®^). L’Ag HBs a été recherché sur les prélèvements de sang veineux par le test rapide HB KIT^®^ de PLETHICO PHARMACEUTICALS LIMITED. Des contrôles de qualité ont été réalisés sur chaque 10^ème^ prélèvement par le Laboratoire du Centre Départemental de Transfusion Sanguine par la méthode ELISA (Enzyme Linked Immuno Sorbent Assay) avec le réactif Monolisa HBs Ultra de BIO RAD^®^ pour le VHB et VIRONOSTIKA pour le VIH.

**Variables étudiées:** L’évènement principal était l’infection par le VHB (oui ou non) et l’exposition principale était l’infection au VIH (oui ou non). Les autres variables étudiées étaient: l’âge, le sexe, la situation matrimoniale, le département d’origine et le niveau d’instruction.

**Traitement et analyse des données:** Les données régulièrement vérifiées et corrigées ont été enregistrées et traitées à l’aide du logiciel EpiInfo^®^ 3.5.1. L’anonymat a été assuré par l’utilisation du numéro d’enregistrement du sujet. Les fréquences ont été estimées avec leurs intervalles de confiance et les moyennes avec leurs écarts-types. L’analyse du rapport entre la prévalence du VHB et les différentes variables indépendantes a été faite par la régression logistique en univariable, puis en multivariable au seuil de significativité de 5%. En analyse univariable, le sexe, l’âge, l’origine géographique et le niveau d’étude ont été chacun croisés avec la variable « hépatite B » (oui, non). Ces 4 variables ont été prises en compte pour une analyse bi-variable en combinaison avec le statut VIH afin de déterminer leur rôle dans la relation entre le VIH et l’hépatite B dans l’échantillon. Pour ce faire, 2 modèles de régression logistique exprimant la prévalence de l’hépatite B en fonction du VIH et de chaque variable ont été comparées: M2 ou modèle ajusté sur chacune des autres variables indépendantes et M3 ou modèle ajusté sur chacune des autres variables indépendantes avec inclusion d’un terme d’interaction entre la variable et le VIH afin d’identifier d’éventuelles modifications d’effet. En absence de modification d’effet, la variation relative (VR) entre le rapport de Prévalence brut de la variable VIH et le RP ajusté (M2) pour les éventuels facteurs de confusion au seuil VR =10%. Un seuil de p < 0,05 a été retenu pour comparer les log-vraisemblance de ces différents modèles.

**Considérations éthiques:** Cette étude a été conduite dans le respect des règles éthiques et de l’accord d’Helsinki. De même, tous les bilans réalisés étaient gratuits pour tous les sujets et les résultats rendus, suivis d’une référence pour la prise en charge appropriée.

## Résultats

**Figure 1:**
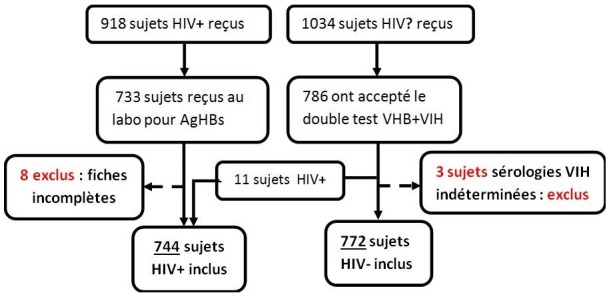
Diagramme de flux de l’étude comparative de la prévalence de l’hépatite B en fonction du statut VIH à Parakou au Bénin, 2012

**Figure 2:**
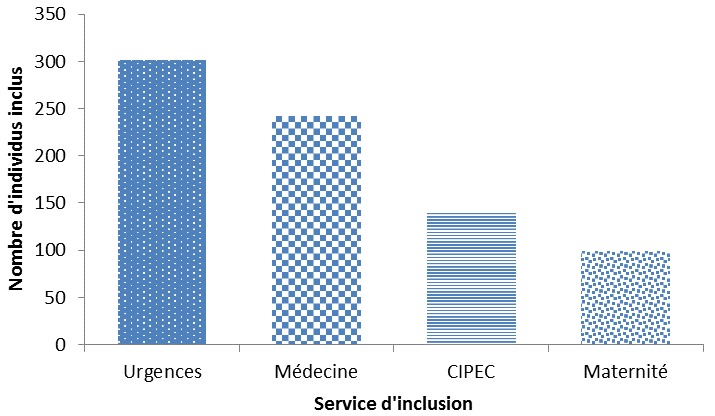
Répartition des 786 sujets de statut VIH inconnu selon le service d’inclusion. Etude sur la prévalence de l’hépatite B à Parakou, 2012

Pendant la période d’étude, 918 personnes séropositives au VIH ont été reçues contre 1034 personnes de statut sérologique VIH inconnu. Le taux de participation a été respectivement de 80,7% pour les sujets séropositifs au VIH et 76,0% pour les sujets de statuts sérologiques VIH inconnus. Le processus d’échantillonnage est décrit dans un diagramme de flux (Figure 1). La quasi-totalité des sujets séropositifs (n = 733) a été recrutée dans le service de Médecine. Le service des Urgences venait en tête dans le recrutement des sujets de statut VIH inconnu au début de l’étude (Figure 2). **Au total 1516 sujets ont été inclus dans l’étude dont 744 sujets séropositifs au VIH.**

### Description de l’échantillon

On dénombrait 987 femmes (65,1%). Il s’agissait d’une population d’adultes jeunes : âge moyen était de 31,1 + 11,1 ans et un intervalle interquartile de 23 à 38 ans. Les sujets originaires du Borgou/Alibori étaient les plus représentés (28,8%), ainsi que ceux ayant arrêté leurs études au secondaire (38,2%). Cependant, les caractéristiques sociodémographiques étaient inégalement réparties entre les sujets séropositifs au VIH et les sujets séronégatifs: les femmes, les plus jeunes, les moins instruits, les autochtones (originaires du Borgou/Alibori) étaient significativement plus représentés parmi les personnes séropositives au VIH (Tableau 1).

**Tableau 1:** Caractéristiques sociodémographiques des 1516 sujets en fonction du statut sérologique VIH: étude sur la prévalence de l’hépatite B à Parakou en 2012

		Séronégatif au VIH		Séropositif VIH		
Var. qualitatives		Effectif (n=772)	%	Effectif (n=744)	%	p
**Sexe**						**<0,0001**
	Féminin	432	56,0	555	65,1	
	Masculin	340	44,0	189	34,9	
**Age (année)**						**<0,0001**
	[15-25[	439	56,9	75	10,1	
	[25-35[	196	25,4	300	40,3	
	[35-45[	73	9,4	237	32,0	
	>45	64	8,3	131	17,6	
**Département d’Origine**						**<0,0001**
	Borgou/Alibori	176	22,8	261	35,0	
	Atacora/Donga	92	12,0	176	23,7	
	Zou/Collines	245	31,7	171	23,0	
	Autres	259	33,5	136	18,3	
**Niveau d’instruction**						**<0,0001**
	Non scolarisé	46	5,9	238	32,0	
	Primaire	60	7,8	218	29,3	
	Secondaire	331	42,9	248	33,3	
	Supérieur	335	43,4	40	5,4	

### Prévalence de l’hépatite B en fonction du VIH

L’antigène de surface du virus de l’hépatite B (AgHBs) a été retrouvé chez 210 sujets sur les 1516 inclus, soit une prévalence globale de 13,9% [IC95%: 12, 2%-15, 7%]. Cette prévalence brute était plus élevée chez les sujets séropositifs au VIH (16,9% ; [IC95%: 14, 3%-19, 9%]) que chez les sujets séronégatifs au VIH (10,9%; [IC95%: 8, 8%-13, 3%]). Le rapport de prévalence brut des sujets séropositifs par rapport aux sujets séronégatifs au VIH a été estimée à 1,67 [IC95%: 1,24-2,25] (p < 0,001).

### Analyse du rôle de tiers facteurs dans la relation VHB/VIH

En analyse univariable, le sexe, l’âge, l’origine géographique et le niveau d’étude ont été chacun significativement lié à la prévalence de l’hépatite B (Tableau 2). L’âge a été identifié comme un facteur de confusion dans la relation entre la prévalence de l’hépatite B et l’infection à VIH: il a été décidé de son maintien dans le modèle final et les résultats du Rapport de Prévalence (RP) de l’infection à VIH dans le modèle final ont été exprimés dans chaque tranche d’âge. De même, l’origine géographique a été identifiée comme un facteur modificateur d’effet de l’infection à VIH sur la prévalence de l’hépatite B. Par contre le sexe et le niveau d’étude n’ont été ni facteur modificateur d’effet, ni facteur de confusion pour l’analyse bivariable.

**Tableau 2:** Déterminants de l’hépatite B au CHUD de Parakou en 2012 (n=1516), régression logistique: analyse univariable

		Ag HBs positif/négatif		
Var. qualitatives		RP estimé	IC_95%_ (RP)	p-value
**Sexe**				**0,0323**
	Féminin	1	-	
	Masculin	1,38	[1,02-1,86]	
**Age (année)**				**0,0003**
	[15-25[	1	-	
	[25-35[	1,98	[1,35-2,91]	
	[35-45[	2,33	[1,53-3,53]	
	>45	1,49	[0,89-2,51]	
**Origine géographique**				**0,0001**
	Borgou/Alibori	1	-	
	Atacora/Donga	0,89	[0,60-1,33]	
	Zou/Collines	0,52	[0,35-0,77]	
	Autres	0,44	[0,29-0,67]	
**Niveau étude**				**0,0245**
	Non scolarisé	1	-	
	Primaire	1,73	[1,08-2,76]	
	Secondaire	1,19	[0,77-1,83]	
	Supérieur	0,92	[0,57-1,50]	

Afin de déterminer l’apport spécifique du sexe et du niveau d’étude dans le modèle de régression logistique de la prévalence de l’hépatite B en fonction de l’infection à VIH et décider de garder ou non ces 2 variables dans le modèle final, 4 modèles de regression logistique prenant en compte le VIH, les classes d’âge et les termes d’interaction entre les classes d’âge et le VIH (Modèle A), ont été comparés dans une démarche ascendante, pas à pas à d’autres modèles en y ajoutant successivement le sexe seul (Modèle B), le niveau d’étude seul (modèle C), puis les deux facteurs (Modèle D). Les valeurs des Chi carré de la « log-vraisemblance » ont été comparées en prenant le modèle MA comme référence. Ainsi les modèles B et C ont été jugés meilleurs au modèle A, de même que le modèle C par rapport au D. Toutefois, l’apport du modèle D n’était pas significatif par rapport au modèle B. L’apport de l’ajustement sur le sexe au modèle initial A est plus important que celui de l’ajustement sur le niveau d’instruction. Mais le modèle D a été retenu pour tenir compte de tous les facteurs potentiellement déterminants.

### Prévalence de l’hépatite B après prise en compte des autres facteurs associés

Les résultats du modèle final sont présentés dans le Tableau 3 et le Tableau 4. Pour les sujets originaires du Borgou/Alibori, après ajustement sur le sexe, l’âge et le niveau d’étude, l’infection au VIH est associée à une prévalence élevée de l’hépatite B. Cette prévalence ne varie pas significativement en fonction du statut VIH pour les sujets non originaires du Borgou/Alibori. Après ajustement sur l’âge, le niveau d’étude, le sexe et le statut VIH, les hommes ont une prévalence de l’hépatite B plus élevée que les femmes. De même, les adultes de 25 à 44 ans ont une prévalence plus élevée que les sujets de 15 à 24 ans. Et enfin les sujets ayant achevé leur cursus au primaire ont une prévalence de l’hépatite B plus élevée que les non scolarisés.

**Tableau 3:** Autres déterminants de la prévalence de l’infection au VHB chez 1516 sujets au CHUD de Parakou en 2012. Régression logistique. Analyse multivariable; modèle final

		Ag HBs positif/négatif	
		RP estimé	IC_95%_ (RP)
**Sexe**			
	Féminin	1	-
	Masculin	**1,58**	**[1,13-2,20]**
**Age (années)**			
	Entre 15 et 24 ans	1	-
	Entre 25 et 34 ans	**1,73**	**[1,13-2,65]**
	Entre 35 et 44 ans	**1,73**	**[1,06-2,83]**
	45ans ou plus	1,11	[0,62-1,98]
**Niveau d’étude**			
	Non scolarisé	1	-
	Primaire	**1,86**	**[1,15-3,00]**
	Secondaire	1,44	[0,91-2,27]
	Supérieur	1,36	[0,76-2,45]

**Tableau 4:** Prévalence de l’infection au VHB en fonction du statut VIH chez 1516 sujets au CHUD de Parakou en 2012: régression logistique, analyse multivariable; modèle final

		Ag HBs positif/négatif	
		RP estimé	IC_95%_ (RP)
***Sujets originaires du Borgou/Alibori***			
**Statut VIH**	Positif vs Négatif	**2,02**	**[1,15-3,55]**
***Sujets originaires de l’Atacora/Donga***			
**Statut VIH**	Positif vs Négatif	0,65	[0,32-1,31]
***Sujets originaires du Zou/Collines***			
**Statut VIH**	Positif vs Négatif	1,01	[0,51-2,00]
***Sujets originaires des autres localités du Bénin et les étrangers***			
**Statut VIH**	Positif vs Négatif	1,75	[0,82-3,74]
**Sexe**			
Féminin vs Masculin		**1,58**	**[1,13-2,20]**
**Age (années)**			
[25-35[vs [15-25[		**1,73**	**[1,13-2,65]**
[35-45[vs [15-25[		**1,73**	**[1,06-2,83]**
>45 vs [15-25[		1,11	[0,62-1,98]
**Niveau d’étude**			
Primaire vs Non scolarisé		**1,86**	**[1,15-3,00]**
Secondaire vs Non scolarisé		1,44	[0,91-2,27]
Supérieur vs Non scolarisé		1,36	[0,76-2,45]

## Discussion

La présente étude a été en partie motivée par l’existence de données initiales de prévalence élevée chez les personnes séropositives [7]. Le type transversal de l’étude est un facteur limitant pour aborder la notion de causalité qui n’entrait pas dans les objectifs de la présente étude. La période de collecte de données a été plus longue pour les sujets séropositifs (Avril 2011 à Juin 2012, soit 15 mois) contre 6 mois pour les sujets à statut VIH inconnu. Ce choix orienté par la collecte préalable des données chez les sujets séropositifs au VIH a pu être source de biais de sélection. Pour les limiter, l’échantillonnage a été exhaustif sur la période d’étude pour chaque cible, ce qui a permis d’obtenir des taux de participation élevés.

Une inégale répartition des caractéristiques sociodémographiques a été notée entre les 2 groupes. Ceci peut être le fait des biais de sélection évoqués précédemment. Mais elle peut aussi refléter une réelle différence entre les populations séropositives et les populations séronégatives. En effet, les facteurs sociologiques, la féminisation de l’infection à VIH en Afrique avec 59% de femmes séropositives sur le total de l’Afrique subsaharienne [9] pourraient expliquer la prédominance des femmes chez les sujets séropositifs. De même, la forte représentation des populations du Borgou/Alibori, suivi de celles des départements limitrophes (Zou/Collines et Atacora/Donga) chez les séropositifs peut s’expliquer par le statut de centre de référence du CHUD de Parakou qui reçoit les populations des départements limitrophes. Le jeune âge des sujets recrutés séronégatifs par rapport aux sujets séropositifs est dû à la moyenne d’âge très basse des candidats au dépistage volontaire et des sujets reçus aux urgences. Le 1^er^ était un centre d’information fréquenté surtout par la jeunesse, et le service des Urgences recueillant un grand nombre de blessés par accidents de la voie publique dont majoritairement des jeunes [10]. Le dépistage de l’hépatite B a été fait par la recherche de l’Ag HBs pour tenir compte du plateau technique et des moyens disponibles. Ceci n’est pas conforme aux recommandations qui proposent d’associer la recherche des anticorps anti-HBc surtout chez les sujets infectés par le VIH [11]. Cependant, l’Ag HBs a été le plus souvent été utilisé [12–15] malgré le risque de sous-estimation de la prévalence de l’hépatite B lié aux hépatites B occultes estimés à environ 5% des cas d’hépatite B [16].

La prévalence de l’hépatite B chez les sujets séropositifs au VIH dans notre étude était comparable aux estimations d’une métaanalyse incluant 60 publications en Afrique en 2010 [3] et à la plupart des estimations faites dans les pays africains subsahariens pour les patients séropositives [17,18], mais plus élevée que celle retrouvée dans le nord de l’Afrique et sur les autres continents [13,18–20]. De même, la prévalence de l’hépatite B chez les sujets séronégatifs au VIH est comparable aux estimations de l’OMS pour l’Afrique Sub-Saharienne, soit entre 5 et 10 % de la population adulte [6].

Après prise en compte des potentiels tiers facteurs, une différence significative de la prévalence de l’hépatite B par rapport au VIH n’a été mise en évidence que dans le sous-groupe des personnes originaires du Borgou et de l’Alibori. Dans cette sous-population, le VIH est associé à une prévalence plus élevée de l’hépatite comme le suggère les données de la littérature [3,7]. Nous avons identifié l’origine géographique comme un facteur modificateur de l’effet de l’infection à VIH sur la prévalence de l’hépatite B. Ceci corrobore la variabilité de cette prévalence à travers les espaces, mais aussi en fonction du type de population. Mais la différence peut également provenir d’une non représentativité des sujets d’autres origines en raison de leur sous-représentation dans le groupe des sujets séropositifs. L’origine géographique devrait donc être prise en compte comme facteur de randomisation lorsque l’on étudie la prévalence de l’hépatite B en fonction du VIH.

Après ajustement, les sujets de 25 à 44 ans ont eu une prévalence de l’hépatite B plus élevée par rapport aux plus jeunes de 15 à 24 ans dans notre étude. Au Brésil en 2012, Aires et al. avait identifié que l’âge ? 50 ans était associé à une prévalence plus élevée de l’hépatite B [21]. Ce ne fut pas le cas dans notre échantillon, mais ce résultat suggère une prévalence plus élevée en fonction de l’âge. Les données de cette étude ont également montré une plus grande exposition des hommes par rapport aux femmes pour l’hépatite B après ajustement sur les autres facteurs. Le mécanisme de cette surexposition est à rechercher. Enfin les sujets ayant achevé leur cursus au primaire ont une prévalence de l’hépatite B plus élevée, ce qui est contraire aux idées reçues car il est admis communément que l’accès à l’éducation améliore les connaissances pour une meilleure santé [22].

## Conclusion

Cette étude complète les données existantes et permet de comparer la prévalence de l’infection à VIH en fonction du statut sérologique VIH en milieu hospitalier à Parakou et d’identifier des hypothèses à vérifier par des études plus élaborées pour approfondir la thématique. Cette étude confirme également une prévalence plus forte de l’hépatite B chez les personnes porteuses du VIH comparativement aux séronégatives. La prévalence de la co-infection par l’hépatite B et le VIH serait associée à l’origine géographique, au sexe, à l’âge et au niveau d’instruction. Cette co-infection crée un double fardeau qui suggère une intégration des programmes de lutte contre ces deux fléaux afin d’optimiser les ressources et les résultats.

### Etat des connaissances actuelles sur le sujet

- La prévalence de l’infection au virus de l’hépatite B est élevée au Bénin.

### Contribution de notre étude à la connaissance

- La prévalence de l’hépatite B est plus élevée chez les personnes séropositives au VIH que chez les personnes séronégatives;
- La prévalence de co-infection VIH/VHB varie significativement selon le sexe, l’origine géographique et le niveau d’instruction.

## Conflits d’intérêts

Les auteurs ne déclarent aucun conflit d'intérêts.
